# Maternal sevoflurane exposure induces neurotoxicity in offspring rats *via* the CB1R/CDK5/p-tau pathway

**DOI:** 10.3389/fphar.2022.1066713

**Published:** 2023-01-10

**Authors:** Yuxiao Wan, Ziyi Wu, Xingyue Li, Ping Zhao

**Affiliations:** Department of Anesthesiology, Shengjing Hospital of China Medical University, Shenyang, China

**Keywords:** CB1R, learning and memory, hippocampus, rimonabant, sevoflurane, tau

## Abstract

Sevoflurane is widely used for maternal anesthesia during pregnancy. Sevoflurane exposure of rats at mid-gestation can cause abnormal development of the central nervous system in their offspring. Sevoflurane is known to increase the expression of cannabinoid 1 receptor (CB1R) in the hippocampus. However, the effect of cannabinoid 1 receptor on fetal and offspring rats after maternal anesthesia is still unclear. At gestational day 14, pregnant rats were subjected to 2-h exposure to 3.5% sevoflurane or air. Rats underwent intraperitoneal injection with saline or rimonabant (1 mg/kg) 30 min prior to sevoflurane or air exposure. cannabinoid 1 receptor, cyclin-dependent kinase 5 (CDK5), p35, p25, tau, and p-tau expression in fetal brains was measured at 6, 12, and 24 h post-sevoflurane/air exposure. Neurobehavioral and Morris water maze tests were performed postnatal days 3–33. The expression of cannabinoid 1 receptor/cyclin-dependent kinase 5/p-tau and histopathological staining of brain tissues in offspring rats was observed. We found that a single exposure to sevoflurane upregulated the activity of cyclin-dependent kinase 5 and the level of p-tau *via* cannabinoid 1 receptor. This was accompanied by the diminished number of neurons and dendritic spines in hippocampal CA1 regions. Finally, these effects induced lower scores and platform crossing times in behavioral tests. The present study suggests that a single exposure to 3.5% sevoflurane of rats at mid-gestation impairs neurobehavioral abilities and cognitive memory in offspring. cannabinoid 1 receptor is a possible target for the amelioration of postnatal neurobehavioral ability and cognitive memory impairments induced by maternal anesthesia.

## Introduction

In recent years, the mass emergence of fetal treatments, intrauterine diagnoses, and minimally invasive technologies has increased the numbers of women receiving surgical treatments during pregnancy. This has triggered a sharp elevation in women exposed to anesthetics at mid-gestation (Eskandari and Alipour, 2020). Due to their solubility and pharmacokinetics, anesthetics may easily pass through the placental barrier, resulting in a risk of fetal exposure to anesthetics (Reitman and Flood, 2011). Of note, the mid-gestation is an important period of fetal neural development and neuron migration, during which the nervous system is particularly susceptible to the external environment. Clinical research has reported that general anesthesia exposure may elicite the development of abnormal behaviors and learning ability in postnatal fetuses (Creeley and Olney, 2010; Sun, 2010). Sevoflurane is the most widely used inhaled anesthetic and is often used in pregnant women. In 2016, the US Food and Drug Administration announced that repeated or prolonged operations on fetuses and infants may lead to neurotoxicity related to exposure to anesthetics (Stockwell, 2017). Our previous research has shown that repeated exposure to 3% sevoflurane or a single exposure to 3.5% sevoflurane of pregnant rats induced long-term learning and memory impairment in their neonatal rats (Wang et al., 2018; Wu et al., 2018). Therefore, this study continues this exploration. Here, we explored the molecular mechanisms of memory impairments in neonatal rats subsequent to maternal exposure to 3.5% sevoflurane during pregnancy.

Cannabinoid receptor 1 (CB1R) is highly expressed in the presynaptic membrane of nerve endings and assumes a pivotal role in regulating neurotransmitter release, nerve development, synapse formation, learning, and memory (Cristino et al., 2020). Neurodegenerative diseases, such as Alzheimer’s disease, multiple sclerosis, and Huntington’s disease, are linked to the aberrant expression of CB1R (Aymerich et al., 2018). The pathophysiological manifestations and clinical symptoms of anesthesia-induced neurological abnormalities are similar to these neurodegenerative disorders. However, it is still unclear whether CB1R is involved in sevoflurane anesthesia-related memory impairments.

CB1R can mediate the activation of cyclin-dependent kinase-5 (CDK5) signal transduction, delay synapse maturation, and arouse neurobehavioral deficits (Joshi et al., 2019). CDK5 activity is orchestrated by lipid associated membrane protein (p35) and its cleavage product (p25), and a change in p25/p35 ratio reflects this activity. *In vitro* experiment showed that a sevoflurane-induced increase in CDK5 blocks the dendritic development of cortical neurons in rats (Liao et al., 2021). Additionally, specific inhibition of CDK5 ameliorates the symptoms of cognitive impairment in sevoflurane-stimulated neonatal rats (Liu et al., 2017). Tau protein is involved in facilitating the assembly of microtubules and is essential for maintaining the function, growth, and development of neurons (Stoothoff and Johnson, 2005). Numerous studies have established that the increase in CDK5 activity can cause the hyperphosphorylation of tau (p-tau), responsible for the destruction of neuronal functions (Xiao et al., 2018).

Therefore, the present study explores changes in the learning and memory of neonatal rats when maternal rats are exposed to 3.5% of sevoflurane at mid-gestation and investigates whether the CB1R/CDK5/tau pathway participates in this process.

## Materials and methods

### Animals

All Sprague Dawley rats were fed in a room at 23°C ± 1°C with a 12-h/12-h light/dark cycle and water and food *ad libitum*. All experiments were implemented under the ratification from the Laboratory Animal Care Committee of China Medical University (P2019PS198K). All procedures followed NIH Guidelines for the Care and Use of Laboratory Animals. Every effort was taken to minimize the number of used rats and their suffering.

### Experimental design and treatment groups

**FIGURE 1:**
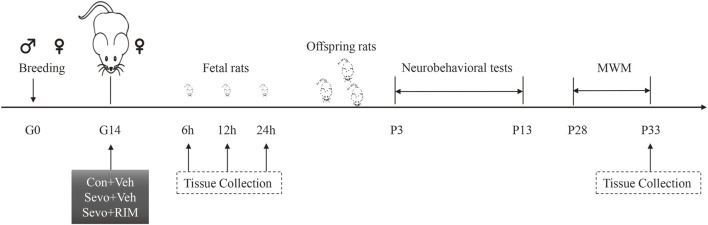
Schematic timeline of this research. Pregnant rats received 2-h exposure of 3.5% sevoflurane or air in the day 0 of gestation (G0). Thirty min prior to sevoflurane or air exposure, rats were subjected to intraperitoneal injection with Rimonabant (RIM, 1 mg/kg) or vehicle (Veh). Brain tissues were harvested from fetal rats at 6, 12, or 24 h subsequent to sevoflurane exposure. The offspring rats in each group were employed for neurobehavioral tests and Morris water maze (MWM) tests during P3–33. Next, the hippocampus was harvested 1 h after MWM test.

The experimental protocol is shown in Figure 1. One male rat was caged with three female rats for mating. The female rats were thought to be in day 0 of gestation (G0) if sperm or vaginal emboli was detected. In total, 36 pregnant rats were randomly allocated into controls + vehicle (Con + Veh), sevoflurane + vehicle (Sevo + Veh), and sevoflurane + rimonabant, a selective antagonist of CB1R (Sevo + RIM) groups (*n* = 12 rats per group). 10 mg RIM was dissolved into 500 µL 5% DNSO to make stock solution. 10 µL stock solution was added into 80 µL 40% PEG300, 10 µL 5% Tween 80 and 100 µL saline. Vehicle solution was prepared as mentioned above, but without RIM. On G14, rats in the Sevo + Veh and Sevo + RIM groups were exposed to 3.5% sevoflurane with 30% oxygen in a specially constructed plastic chamber for 2 h. Animal facility rearing with 30% oxygen only was performed on control animals (Con + Veh) (Wang Y et al., 2018; Li XY et al., 2017). A solution of saline or rimonabant (1 mg/kg) was injected intraperitoneally 30 min before oxygen or sevoflurane exposure, respectively. The dose selection of rimonabant was based on previous reports (Shivakumar et al., 2020) and our preliminary study. Cesarean sections (3 dams/group at each time point) were conducted at 6, 12, or 24 h after modeling. The fetal brains in each group (six offspring rats, two pups/dam) were extracted for molecular study. The remaining dams (*n* = 3 per group) were allowed to deliver naturally. The behavioral testing was performed on twelve offspring rats from each group (four pups/dam) during postnatal days P3–33. The brain tissues were collected 1 h after the Morris water maze (MWM) test.

### Behavioral tests

Neonatal neurobehaviors can be used as an early sensitive index to reflect the functional development of brain in newborn. It is reported that the behavioral outcomes in the present study mature in neonatal rats after P7 (George et al., 2015).

### Surface righting reflex

To evaluate the coordination of the offspring rats (pups), they were placed on their backs on a horizontal table. The time required for flipping to right themselves in position was recorded. This time was measured three times a day during P3–7 for each pup (Naik AA et al., 2015; Zhang Y et al., 2010; McDonnell-Dowling K et al., 2014).

### Negative geotaxis reflex

The vestibular and proprioceptive functions of pups were evaluated by placing them on a 30° mesh incline in a head-down position. The time taken for pups to turn 180° to an upright position was recorded for a maximum of 60 s. Each pup performed this test three times a day during P3–7 (Naik AA et al., 2015; Zhang Y et al., 2010; McDonnell-Dowling K et al., 2014).

### Forelimb grip test

This test was performed to evaluate the forelimb sensation, muscle strength, and reactivity of the pups. The pups grasped the horizontal rod of a suspension hanger (.5 cm in diameter) using their forelimbs on P7, 9, 11, and 13. The time duration when each pup was able to remain suspended before falling down to the sponge below was measured three times in 1 day and the average value was calculated (McDonnell-Dowling K et al., 2014; Adedara IA et al., 2017).

### Cliff avoidance test

To evaluate the integration of exteroceptive input and locomotor output during P3–7, pups were put on a table with their head and forepaws over the edge. A positive result was recorded if the pups retreated or turned around, otherwise it was negative. The total observation time was 60 s and the observed data reflected the rate of positive responses (Naik AA et al., 2015; Khalki L et al., 2013).

### Sensory reflexes

To evaluate the development of sensory ability, including ear-edge reflex and eyelid reflex, the eyelid and ear edge of the pups was touched with a sterile cotton swab during P6–13. If the corresponding reflex appeared, the trial was deemed positive. The results were expressed as the positive rate of each group (Horvath G et al., 2013).

### MWM test

This behavioral test was conducted as documented in our previous research (Fan et al., 2021). Briefly, the maze (height: 60 cm, diameter: 180 cm) was divided into four quadrants. In the center of the second quadrant, a circular platform was submerged to a depth of 1.5 cm underneath the water surface. During P28–32, all rats completed four sessions each day attempting to locate the hidden platform. Each rat was allowed to swim 90 s without the platform On P33. A Noldus Ethovision XT video analysis system (Netherlands) was adopted for recording escape latency, the times of crossing the platform, and time spent in each quadrant.

### Nissl staining

The procedures for this evaluation were performed as in our previous study. Briefly, three pups in each group were chosen for perfusion with saline and 4% paraformaldehyde. Afterwards, the whole brain of pups were collected and placed into 4% paraformaldehyde overnight at 4°C. After paraffin embedding, 4-µm-thick coronal slices were subjected to Nissl staining. Pyramidal cell layers in the CA1 area of the brain were photographed with a digital microscope. Nissl-positive neurons were counted using the ImageJ software.

### Golgi staining

Golgi staining was carried out following the protocols of the FD rapid GolgiStain™ kit (#pk401A, FD NeuroTechnologies, Inc. Columbia, MD, United States); the rats (3 rats per group) underwent transcardial perfusion of phosphate buffered saline (PBS), and then, their brains were collected and fixed in kit solutions A and B for 2 weeks. Thereafter, brain tissues were transferred to kit solution C for 72-h incubation. The staining was conducted on a 150-µm-thick brain section using kit solutions D and E. As a final step, spine density was tested for each neuron using ImageJ software (number of spines per 10 m).

### Immunofluorescence staining

The rats (3 rats in each group) received transcardial perfusion with PBS before 4% paraformaldehyde treatment. Post-fixation with 4% paraformaldehyde and dehydration in 30% sucrose were performed at 4°C for 24 h. Ten-micron-thick coronal sections of the hippocampus were obtained with a cryostat. Samples were probed with primary antibodies against CBR1 (1:1000, #93815, CST, Danvers, MA, United States), CDK5 (1:1000, #14145, CST), and Nestin (1:200, #11306, Abcam, Cambridge, England). After 24 h, samples were incubated with secondary antibodies. Nuclei were counterstained with DAPI stain solution (#AR1177, Boster Biological Technology, Pleasanton, CA, United States).

### Western blotting

Western blotting was performed on fetal brain tissue and offspring hippocampus according to the method as previously described by our research group (Wu ZY et al., 2018; Li XY et al., 2017). The whole brains of fetuses were removed immediately when the female rats were under cesarean section. Hippocampus of offspring rats was harvested at P33 after the behavior tests. Fetal brain tissue and hippocampus homogenates (*n* = 3 per group) were prepared for determination of CB1R, CDK5, p35, p25, tau, p-tau, and GAPDH levels. Protein levels were examined by incubating hippocampus homogenates with antibodies to CB1R (1:1000, Cell Signaling Technology, 93,815, United States), CDK5 (1:1000, Cell Signaling Technology, 14,145, United States), p35 (1:1000, Cell Signaling Technology, 2,680, United States), p25 (1:1000, Cell Signaling Technology, 2,680, United States), tau (1:1000, Cell Signaling Technology, 46,687, United States), p-tau (1:1000, Cell Signaling Technology, 20,194, United States), and GAPDH (1:1000, Cell Signaling Technology, 2,118, United States). If the target protein is phosphorylation index (tau,p-tau), phosphatase inhibitors should be added in addition to PMSF. Visualization of the bands was performed with enhanced chemiluminescence and quantification was carried out with ImageJ (NIH Image, United States).

### Statistical analysis

All data were manifested as mean ± S.E.M and processed using SPSS 17.0 (SPSS Inc., United States). Additionally, all data underwent one-way ANOVA using Tukey’s *post-hoc* multiple comparison tests that were only conducted if *F* reached a statistical significance of *p* < .05.

## Results

### Maternal sevoflurane exposure triggered impairments in neurobehaviors and cognitive memory in offspring rats

**FIGURE 2:**
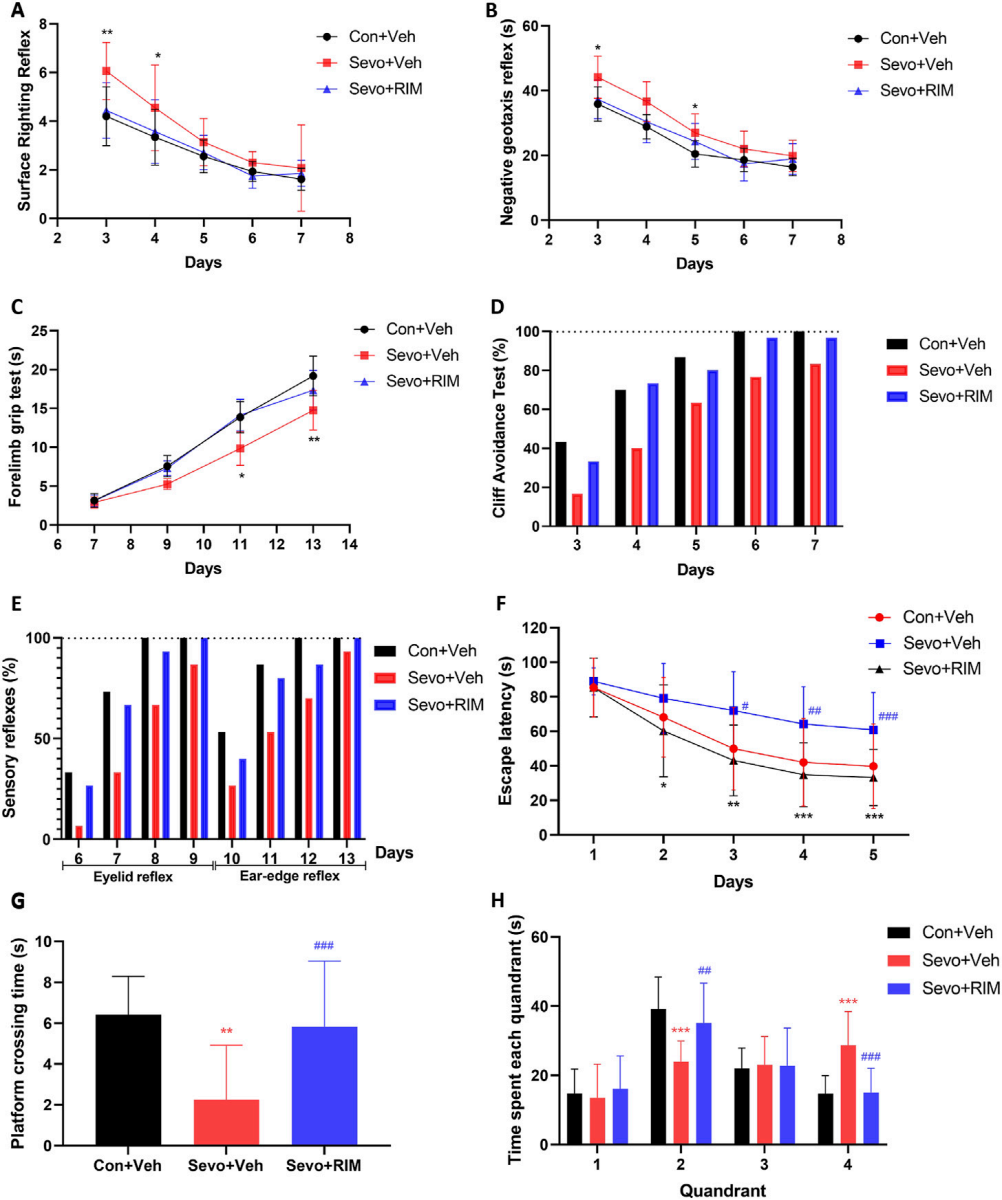
Maternal sevoflurane exposure caused dysfunction in neurobehaviors and cognitive memory in neonatal rats. Neurobehavioral tests (*n* = 12 per group) were performed during P3–13 and included surface righting reflex **(A)**, negative geotaxis reflex **(B)**, forelimb grip test **(C)**, cliff avoidance test **(D)**, and sensory reflexes **(E)**. The MWM tests (*n* = 12 per group) were carried out during P28–33. Escape latency **(F)**, the times of crossing the platform **(G)**, and time spent in each quadrant **(H)** were recorded. Data are depicted as mean ± S.E.M. *p < .05, **p < .01, vs. the Con + Veh group; #*p* < .05, vs. the Sevo + Veh group.

Neurobehavioral and MWM tests were conducted during P3–33 in offspring rats to investigate the function of maternal exposure to sevoflurane in neurological functions and cognitive memory, Figure 2. Compared to the Con + Veh group, rats in the Sevo + Veh group took longer time to turn to right (*p* < .01; Figure 2A) and turn around (*p* < .05; Figure 2B) on P3 and spent shorter time hanging from the suspension hanger in the forearm grip strength test on P13 (*p* < .01; Figure 2C). Cliff avoidance tests or sensory reflexes were not different between the Con + Veh and Sevo + Veh groups (Figures 2D, E). Escape latency of rats were longer in the Sevo + Veh group than in the Con + Veh group for the MWM test during P30–32 (*p* < .05; Figure 2F), accompanied by fewer times of crossing the platform (*p* < .01; Figure 2G) and shorter time in the second quadrant on P33 (*p* < .01; Figure 2H).

RIM pretreatment markedly shortened the time for pups to right themselves (*p* < .05; Figure 2A) and prolonged the time they were able to grasp the hanger (*p* < .05; Figure 2C) versus the Sevo + Veh group. Additionally, in the MWM test, treatment with RIM decreased the escape latency during P30–32 (*p* < .05; Figure 2F) and enhanced the times of crossing the platform (*p* < .05; Figure 2G) and time in the second quadrant on P33 (*p* < .05; Figure 2H) in contrast to the Sevo + Veh group.

### Impacts of maternal sevoflurane exposure on neuronal and dendritic spine densities in the hippocampal CA1 area of neonatal rats

**FIGURE 3:**
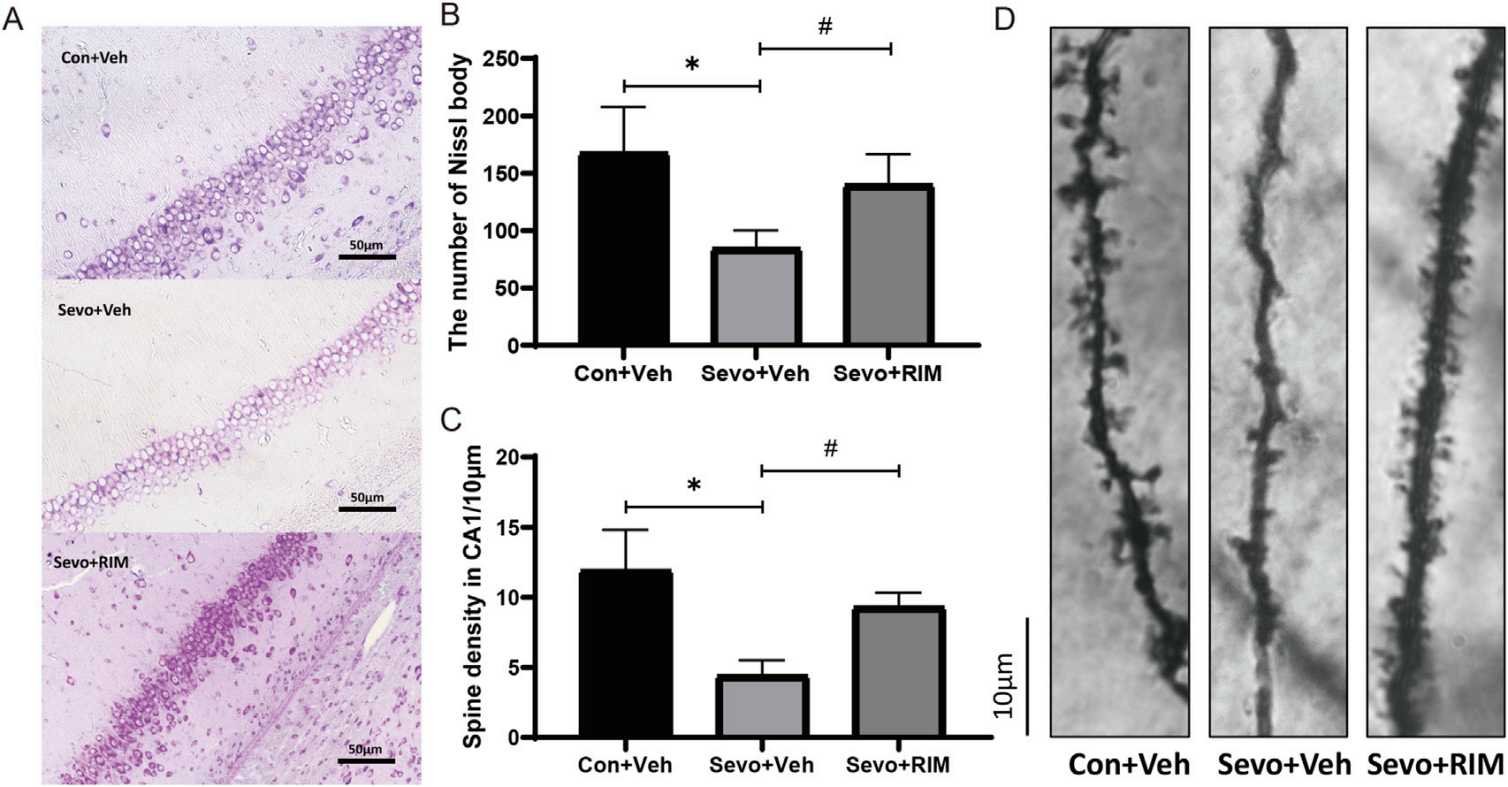
Functions of maternal sevoflurane exposure in neuronal density and dendritic spine density in the hippocampal CA1 area of neonatal rats. Nissl staining under ×400 **(A)** and neuronal density ratio changes **(B)** in each group (*n* = 3). Golgi staining under ×1000 **(D)** and histograms of spine density/10 µm **(C)** in each group (n = 3). Data are displayed as mean ± S.E.M. **p < .01, vs. the Con + Veh group; #*p* < .05, ###*p* < .001, vs. the Sevo + Veh group.

Sevoflurane exposure dramatically decreased the number of neurons (*p* < .05; Figures 3A,B) and dendritic spines (*p* < .01; Figures 3C,D) in the hippocampal CA1 areas in offspring rats of the Sevo + Veh group relative to the Con + Veh group. RIM pretreatment significantly increased the number of neurons (*p* < .001; Figures 3A,B) and dendritic spines (*p* < .05; Figures 3C,D) in the hippocampal CA1 areas of offspring rats belonging to the Con + Veh group versus the Sevo + Veh group.

### Sevoflurane upregulated p-tau expression in the fetal brains *via* the CB1R/CDK5/p25 pathway

**FIGURE 4:**
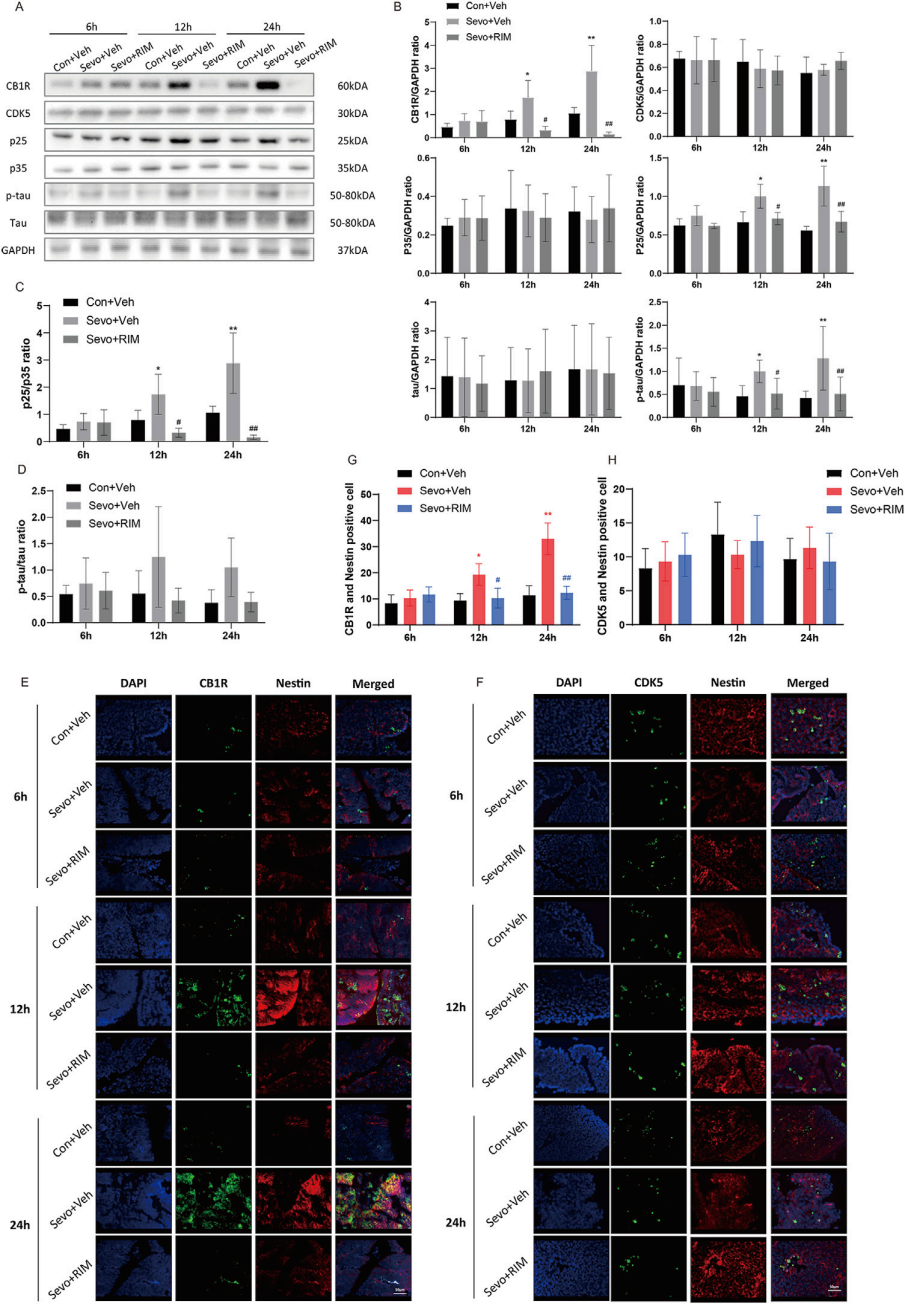
Sevoflurane upregulated p-tau in the fetal brains *via* the CB1R/CDK5/p25 pathway. Assays of western blotting (*n* = 3) demonstrating the expression of CB1R, CDK5, p35, p25, tau, and p-tau **(A,B)** and the value of p25/p35 **(C)** and p-tau/tau **(D)**. Immunofluorescence double staining of CB1R and Nestin (**E**, *n* = 3) and CDK5 and Nestin (**F**, *n* = 3) in the fetal rat brains. Quantification of CB1R/Nestin-positive cells **(G)** and CDK5/Nestin-positive cells **(H)**. Data are manifested as mean ± S.E.M. *p < .05, **p < .01, vs. the Con + Veh group; #*p* < .05, ##*p* < .01, vs. the Sevo + Veh group.

To determine how maternal sevoflurane exposure impairs neurological functions and cognitive memory, we determined CB1R, CDK5, p35, p25, tau, and p-tau expression. As shown in Figures 4A–D, sevoflurane time-dependently increased CB1R expression (12 h, *p* < .05; 24 h, *p* < .01), p25 (12 h, *p* < .05; 24 h, *p* < .01) and tau phosphorylation (12 h, *p* < .05; 24 h, *p* < .01) in the brain of offspring rats of the Sevo + Veh group relative to the Con + Veh group. A higher number of CB1R/Nestin-positive cells (12 h, *p* < .05; 24 h, *p* < .01; Figures 4E,G) were observed in the Sevo + Veh group than in the Con + Veh group. CDK5-, p35-, tau-, and CDK5/Nestin-positive cells did (Figures 4F,H) not differ between the Sevo + Veh and Con + Veh groups. RIM pretreatment negated sevoflurane-stimulated increases in CB1R (12 h, *p* < .05; 24 h, *p* < .01), p25 (12 h, *p* < .05; 24 h, *p* < .0112, 24 h, *p* < .05), and p-tau (12 h, *p* < .05; 24 h, *p* < .01) expression and the number of CB1R/Nestin-positive cells (12 h, *p* < .05; 24 h, *p* < .01).

### Sevoflurane increased p-tau expression in the hippocampus of offspring rats

**FIGURE 5:**
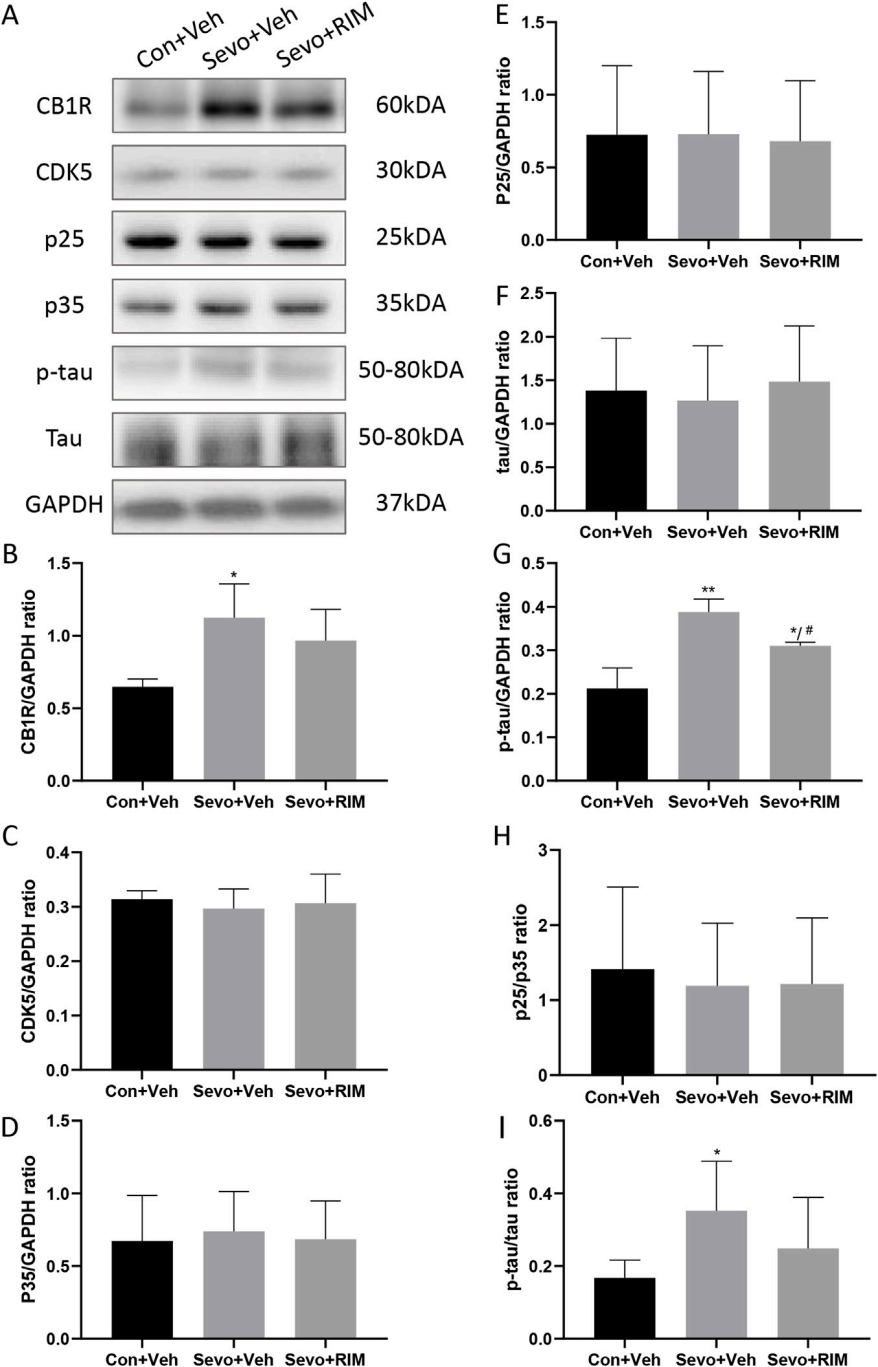
Sevoflurane augmented p-tau expression in the hippocampus of offspring rats. Representative western blots **(A)** and quantitation **(B–I)** of western blotting assays (*n* = 3 rats/group) showing CB1R, p35, CDK5, p25, tau, and p-tau expression in the hippocampus. Data are summarized as mean ± S.E.M. *p < .05, **p < .01, vs. the Con + Veh group; #*p* < .05, vs. the Sevo + Veh group.

To ascertain whether sevoflurane activated the CB1R/p25/p-tau pathway in offspring rats, CB1R, CDK5, p35, p25, tau, and p-tau expression was tested in the hippocampus (Figure 5A). Sevoflurane substantially augmented CB1R (*p* < .05) and p-tau (*p* < .01) expression in the Sevo + Veh group versus the Con + Veh group (Figures 5B,G,I). No considerable alteration was noted in CDK5, p35, p25, and tau expression between the Con + Veh and Sevo + Veh groups (Figures 5C,D,E,F,H). The RIM pretreatment may interfere with sevoflurane’s effects on p-tau expression (*p* < .05).

## Discussion

Our research unveiled that a single exposure of rats at mid-gestation to 3.5% sevoflurane impaired neurobehavioral abilities and long-term cognitive memory accompanied by increased p-tau expression in the hippocampus of offspring rats and fetuses. We first found that rimonabant, a selective antagonist of CB1R, can block the activation of the CB1R/CDK5/p-tau pathway, improve neurobehavioral abilities, and alleviate cognitive memory loss induced by sevoflurane.

Former research has mostly concentrated on the function of sevoflurane in ethology during adolescence or adulthood. The eight arm maze, MWM, Y maze, and open field tests have often been used to evaluate learning and memory of rodents (Xu et al., 2016; Yang et al., 2020). In our study, neurobehavioral tests were used for the first time to comprehensively evaluate the sensory, motor, coordination, and cognitive abilities to accurately reflect the functional development of the brain in offspring rats. Our results showed that the time it took pups in the Sevo + Veh group to right themselves and to turn around was evidently prolonged in the Sevo + Veh group relative to the Con + Veh group on P3. This indicates that coordination, vestibular, and proprioceptive functions were damaged in the offspring rats. In the forelimb grip test, suspension time of the Sevo + Veh group was prominently shortened versus the Con + Veh group on P11 and P13, indicating that the forelimb sensation, muscle strength, and reactivity of offspring rats were impeded subsequent to maternal sevoflurane exposure. Although there were no significant differences in the cliff avoidance and sensory reflex experiments between the two groups, the Sevo + Veh group displayed lower positive rates than the Con + Veh group on the same day. This demonstrated that maternal sevoflurane exposure may decrease the risk assessment ability and sensory functions of offspring rats. The MWM test depicted higher escape latency, fewer times of crossing the platform, and shorter time in the second quadrant on P28–33, suggesting that maternal exposure to sevoflurane resulted in cognitive memory dysfunction in the offspring rats. After exposure to RIM, the behavioral abnormalities were reversed in the Sevo + Veh group. These data demonstrate the crucial involvement of CB1R in maternal sevoflurane exposure-stimulated neurobehavioral and cognitive impairments in the offspring rats.

Tau, belonging to the family of microtubule-associated proteins, participates in cell homeostasis, cell movement, and axon signal transmission (Liu et al., 2015). A previous study has shown the stimulating impacts of sevoflurane anesthesia on p-tau expression and cognitive abnormalities in neonatal mice (Liao et al., 2021). Non-etheless, the impacts of maternal sevoflurane exposure on tau in the offspring rats are still unknown. Our results demonstrated that p-tau (Ser404) expression in the hippocampus of fetal and postnatal rats noticeably increased in the Sevo + Veh group versus the Con + Veh group. Previous studies also reported that cognitive dysfunction caused by sevoflurane was related to the phosphorylation of Ser396/404 epitope of tau protein (Freche et al., 2012; Zhang YH et al., 2021). Tau expression did not change significantly as tau widely exists in the central nervous system under physiological conditions (Richetin et al., 2020). Although a part of tau is hyperphosphorylated, the total amount of tau does not obviously decrease. P-tau expression was markedly augmented in the Sevo + Veh group in contrast to the Con + Veh group on P33, while the upstream expression of CDK5/p25 returned to normal, indicating that the hyperphosphorylation of tau is not easy to reverse rapidly after maternal sevoflurane exposure.

CB1R is located in the presynaptic membrane of nerve terminals and regulates the release of neurotransmitters (Di Marzo and Piscitelli, 2015). Isoflurane treatment of mouse hippocampal neural stem cells leads to the dose-dependent increase in CB1R mRNA levels (Zhou et al., 2016). Repeated sevoflurane exposure increases the levels of endogenous cannabinoid and activates extracellular signal-regulated kinases (Zheng et al., 2015; Zhang et al., 2016). In the current research, the data established that 3.5% sevoflurane could time-dependently upregulate the expression of CB1R in fetal and offspring rats. During the development of synapses, CB1R can mediate the activation of CDK5 and cause neurological deficits (Roufayel and Murshid, 2019). An increase in CDK5 activity can cause the hyperphosphorylation of tau (p-tau) followed by a series of pathological changes of nervous system, such as neuronal injury (Liu et al., 2017). However, the expression of CDK5 did not change in our study. This is because, similar to other CDK family proteins, CDK5 does not have enzymatic activity in a monomeric state except for the binding of its specific regulatory units, like p35 and p25. In addition, its activity can be reflected by the ratio of p35 and p25 (Roufayel and Murshid, 2019). The abnormal activity of CDK5/p25 can elicit neurological abnormalities and a variety of neurodegenerative diseases (Bu et al., 2002; Ba et al., 2017; Zeb et al., 2019). Our data elucidated that p25 in fetal brains, but not in offspring rats, is remarkably upregulated in the Sevo + Veh group relative to the Con + Veh group, suggesting that sevoflurane could activate CDK5/p25 in fetal rats. P25, a cleavage product of p35, possesses longer half-life than p35, which, therefore, can observably prolong the activation of CDK5. This may be the reason why p35 has not changed significantly in the current research.

It was previously elaborated that hippocampal, peripheral, and systemic blockade of CB1R abolishes the stress-stimulated memory abnormalities (Busquets-Garcia et al., 2016). Intra-medial prefrontal cortex or systemic administration of rimonabant enhances extinction of cocaine-stimulated conditioned place preference memory but impairs consolidation of low-dose cocaine-stimulated conditioned place preference memory (Hu et al., 2015). The data obtained from our research demonstrated that 3.5% sevoflurane exposure of maternal rats activated CB1R/CDK5/p25/p-tau pathway in the hippocampus of fetuses and offspring rats, resulting in diminishment in neuron and dendritic spine densities in the hippocampal CA1 area. These alterations indicated neurotoxicity occurrence in offspring rats and the potential role of CB1R pathway in sevoflurane exposure. We also found that a CB1R antagonist could markedly block the activation of the CB1R/CDK5/p25/p-tau pathway. Subsequently, it reversed sevoflurane-induced decrease in neuron density and dendritic spine density in the CA1 region. Finally, it ameliorated neurobehavioral and cognitive impairments in sevoflurane-treated rats. The present study indicates that CB1R critically manipulates sevoflurane-stimulated neurotoxicity in fetal and offspring rats. Therefore, our research provides an important theoretical basis for the development of new drugs targeting CB1R.

There is two limitations in the present study: 1) We did not perform Control + RIM group to evaluate deleterious possible effects in offspring. That is because the toxicological effects of rimonabant in clinic is clear, including tremor, dyskinesia, anxiety and hyperexcitability (Christensen et al., 2007). Data from FDA in the United States showed that rimonabant could increase the risk of suicide of users (Vinod and Hungund, 2006). However, it is necessary to explore the deleterious effects in offspring as well. 2) Tau protein have more than eighty epitopes to be phosphorylated (Freche et al., 2012). However, we only explore the expression of p-tau (Ser404) in sevoflurane-treated rats.

## Data Availability

The raw data supporting the conclusion of this article will be made available by the authors, without undue reservation.
